# Association Between Shift Work and Auditory–Cognitive Processing in Middle-Aged Healthcare Workers

**DOI:** 10.3390/audiolres15060145

**Published:** 2025-10-25

**Authors:** Margarida Roque, Tatiana Marques, Margarida Serrano

**Affiliations:** 1 Polytechnic University of Coimbra, Rua da Misericórdia, Lagar dos Cortiços, S. Martinho do Bispo, 3045-093 Coimbra, Portugal; roquemargarida2003@gmail.com (M.R.); tatiana.marques@estesc.ipc.pt (T.M.); 2 Coimbra Institute for Biomedical Imaging and Translational Research, University of Coimbra, 3000-548 Coimbra, Portugal; 3 Faculty of Medicine, University of Porto, 4200-319 Porto, Portugal; 4 H&TRC—Health & Technology Research Center, Coimbra Health School, Polytechnic University of Coimbra, Rua 5 de Outubro, 3046-854 Coimbra, Portugal

**Keywords:** auditory processing, cognition, healthcare workers, middle aged, shift work

## Abstract

Background/Objectives: Shift work in healthcare professionals affects performance in high cognitive processing, especially in complex environments. However, the beneficial effects that working in complex environments may have on auditory–cognitive processing remain unknown. These professionals face increased challenges in decision-making due to factors such as noise exposure and sleep disturbances, which may lead to the development of enhanced auditory–cognitive resources. This study aims to investigate the associations between shift work and auditory–cognitive processing in middle-aged healthcare workers. Methods: Thirty middle-aged healthcare workers were equally allocated to a shift worker (SW) or a fixed-schedule worker (FSW) group. Performance on a cognitive test, and in pure-tone audiometry, speech in quiet and noise, and listening effort were used to explore whether correlations were specific to shift work. Results: Exploratory analyses indicated that shift workers tended to perform better in visuospatial/executive function, memory recall, memory index, orientation, and total MoCA score domains compared to fixed-schedule workers. In the SW group, hearing thresholds correlated with memory recall and memory index. In the FSW group, hearing thresholds correlated with orientation, memory index, and total MoCA score, while listening effort correlated with naming, and speech intelligibility in quiet correlated with total MoCA scores. Conclusions: These exploratory findings suggest that shift work may be linked to distinct auditory–cognitive patterns, with potential compensatory mechanisms in visuospatial/executive functions and memory among middle-aged healthcare workers. Larger, longitudinal studies are warranted to confirm whether these patterns reflect true adaptive mechanisms.

## 1. Introduction

Healthcare systems exemplify complex environments characterized by numerous interacting and interdependent components. Their nonlinear, dynamic interactions generate emergent properties and unpredictable behavior that cannot be deduced solely from examining individual parts. Overall, the effectiveness of such systems fundamentally relies on complex interactions, such as the contribution of healthcare workers, defined as individuals whose primary purpose is to improve health (i.e., clinical professionals, technicians, and support staff who deliver services including prevention, treatment, and rehabilitation) and the organization of these workers’ distinct schedules [1,2].

Fixed-scheduled workers are typically employed in settings such as outpatient consultations and elective surgery units, whereas shift workers are critical for maintaining continuous 24/7 operations (e.g., emergency medical services). Additionally, shift workers face unique challenges, including fluctuating patient volumes, overcrowding, limited resources, and frequent exposure to traumatic events [3,4]. These occupational demands are further compounded by biological factors. For example, hormone secretion, sleep patterns, alertness, and subjective perception are fundamentally regulated by the endogenous circadian rhythm [5].

In fact, sleep–wake regulation results from two interacting processes, namely the homeostatic process, in which sleep pressure accumulates during wakefulness and dissipates during sleep [6], and the circadian process, which drives endogenous fluctuations in sleep propensity and wakefulness tendency across the 24-h biological cycle [7]. Therefore, when work requires night shift work, a disruption between the circadian system and the external environment may occur, leading to a state of circadian desynchronization comparable to severe jet lag. Tolerance to this disruption varies substantially across individuals, influenced by factors such as gene–environment interactions, chronotype, light/dark exposure patterns, work schedule design, personal sleep/light habits, age, and health status [8]. Indeed, one of the primary complaints, especially among healthcare shift work novices, is insufficient daytime sleep.

Persistent sleep restriction leads to accumulating deficits, resulting in acute or chronic fatigue, excessive sleepiness, and degraded alertness and cognitive performance. These deficits increase errors and accident risks during safety-critical tasks [8,9]. Moreover, chronic misalignment between sleep/wake cycles and circadian timing has been associated with widespread hormonal and metabolic dysregulation. Consequently, shift workers face a substantially elevated risk of developing multiple chronic conditions, including cardiovascular disease, metabolic syndrome, gastrointestinal disorders, various cancers, reproductive issues (e.g., menstrual irregularities and pregnancy complications), and psychological disorders, with risks significantly higher than those observed in fixed-schedule workers [8,10,11].

Several studies have reported deficits in cognitive function among these workers, particularly in attention, alertness, and visual–motor performance, even when waking time is less than 16 h [12,13,14,15]. Additionally, excessive environmental noise in healthcare units detrimentally affects healthcare professionals’ performance [16]. For these professionals, clear hearing is essential to minimize listening effort and preserve shared cognitive resources (e.g., working memory, attention, and language processing), which are critical for complex decision-making and daily professional tasks. Compromised hearing markedly increases cognitive load, as demonstrated by dual-task paradigms revealing reduced performance in concurrent non-auditory tasks during speech-in-noise processing, especially among individuals with hearing loss or those working in acoustically challenging environments [17]. However, to determine if a shift system is detrimental to auditory–cognitive performance, more exhaustive studies should be carried out. 

In fact, exposure to complex, dynamic healthcare environments may provide significant cognitive or auditory–cognitive benefits under high-pressure conditions. Effective decision-making in such complex environments requires sustained engagement of high-order cognitive functions, particularly executive functions such as inhibition, working memory (essential for organizing non-perceptual information and retrieving prior strategies), and cognitive flexibility. These functions are essential to gather information, interpret feedback, test hypotheses, update knowledge, and make evidence-based decisions. Such top-down mechanisms are crucial not only for accurate sound identification but also for sustaining attention, controlling automatic responses, and flexibly adapting to dynamic workplace demands [18,19].

In this context, the purpose of our study was to investigate associations between shift work and auditory–cognitive processing through comparisons of middle-aged healthcare shift workers and fixed-schedule workers.

## 2. Materials and Methods

### 2.1. Ethics

Ethical approval was obtained from the Ethics Committee of the Polytechnic Institute of Coimbra (approval number D51/2024). All participants provided both written and verbal informed consent.

### 2.2. Participants

A total of thirty healthcare professionals were recruited from a tertiary hospital and, according to their work shifts, assigned to either the fixed-schedule work (FSW) group (N = 15), if they had a fixed daytime schedule, or the shift work (SW) group (N = 15), if they worked in shift schedules that included both daytime and nighttime shifts. All participants were native speakers of European Portuguese, right-handed, had a type A or C1 tympanogram in both ears, and were able to read and write autonomously, which constituted the inclusion criteria for the study. Age and employment at a hospital were also inclusion factors. Neuropsychiatric diseases were established as exclusion criteria. Demographic characteristics were similar across groups, with two males (13.3%) and thirteen females (86.7%) in each. Twelve participants (80%) had completed higher education. The SW group had a median age of 52 years (range: 43–61) and a median professional experience of 30 years (range: 3–30), with 11 (73.3%) reporting workplace noise exposure. The FSW group had a median age of 47 years (range: 41–64) and a median professional experience of 26 years (range: 8–41), with 13 (86.7%) reporting workplace noise exposure. 

Regarding healthcare workers’ professional categories, nurses were more frequently represented (57.9%) in the SW group than in the FSW group (42.1%), while healthcare assistants were evenly distributed across groups (50% each) (see Table 1). 

### 2.3. Procedure

All participants completed a questionnaire to characterize the sample demographically and to obtain information on cognitive, clinical, and sleep characteristics. In addition, the Montreal Cognitive Assessment (MoCA; version 7.1; available at https://www.mocatest.org/) was administered, and all participants underwent pure-tone audiometry, a speech intelligibility test in quiet and in noise, and listening effort assessments using the Audiology4All app, version 1.0 (Audiology4All, Lisbon, Portugal). Details regarding the development of the Audiology4All app have been described previously [20]. 

### 2.4. Cognitive Evaluation

Cognitive assessment was carried out using the MoCA, a brief and widely used screening tool with high sensitivity for detecting mild cognitive impairment (MCI) and early dementia. The MoCA evaluates multiple cognitive domains, including attention and concentration, executive function, memory, language, visuospatial abilities, abstract reasoning, and orientation. Administration typically requires approximately 10 to 15 min, with scores ranging from 0 to 30. A validated Portuguese version, adapted for the Portuguese population, was used [21].

### 2.5. Auditory Evaluation

Pure-tone thresholds were obtained by air conduction at 500, 1000, 2000, 4000, 6000, and 8000 Hz and by bone conduction at 500, 1000, 2000, and 4000 Hz, using a GSI Audiostar Pro audiometer (Grason-Stadler, Eden Prairie, MN, USA) with Radioear DD 65 v2 headphones (Radioear, New Eagle, PA, USA). Pure-tone average (PTA) was calculated for each ear using the mean air conduction thresholds at 500, 1000, 2000, and 4000 Hz. Additionally, the average air conduction thresholds in 2000, 4000, and 6000 Hz were determined for each ear to specifically assess high-frequency hearing, which plays a crucial role in speech intelligibility. 

Subsequently, an intelligibility test in quiet was performed using two lists of twenty-five phonetically balanced disyllabic words, presented in free field at 65 dB SPL. Participants were seated at the center of a 2 × 3 m sound-treated booth, with stimuli delivered from two Wharfedale speakers (Wharfedale, Huntingdon, Cambridgeshire, UK) positioned at 135° and 225° and connected to a GSI Audiostar Pro audiometer. Average performance across the two lists presented was calculated. 

Finally, participants completed both the intelligibility in noise test and the listening effort assessment using the Audiology4All app [20]. The speech-in-noise test uses four phonetically balanced word lists presented at a zero dB signal-to-noise ratio. Only one list is displayed in each app view. Each trial consisted of one target word followed by a four-alternative forced-choice recognition task. Performance was expressed as the percentage of correctly identified words across 16 trials. [20]. 

Listening effort was evaluated using a dual-task paradigm implemented in the Audiology4All app. Word lists were presented at a +5 signal/noise ratio after being randomly generated from a pool of sixty-eight disyllabic words. The primary task was word recognition in noise, and the secondary task was recalling words. The Audiology4All app generates word lists of varying lengths, with each test comprising three lists. After each list, participants were shown nine words and asked to identify the last three presented. The score was the percentage of correct responses for the antepenultimate (third-from-last) word across the three lists. The antepenultimate word’s recall acts as a metric for residual cognitive capacity. Lower scores indicated more effort for listening, serving as an index of residual cognitive capacity [20]. All verbal stimuli were presented in a female voice. 

### 2.6. Statistical Analysis

Descriptive statistics were used to summarize the data. Continuous variables are reported as mean, median, and standard deviation (SD), and categorical variables as counts (N) and percentages (%). Associations between variables were examined using Spearman’s rank correlation. Group comparisons were performed with the Mann–Whitney U test. Statistical significance was set at *p* < 0.05.

## 3. Results

Among the SW group, 33.3% of the participants had reported a history of otitis, 6.7% reported vertigo, and 53.3% were on medication. Comorbidities included elevated cholesterol (11.1%), cholesterol with hypertension (22.2%), and isolated cases of more complex conditions such as diabetes, hyperthyroidism, sleep apnea, and obesity. Median sleep duration was 6 h (range: 4–8), decreasing to 3 h (0–7) on the night before assessment. Forty percent reported good rest, 66.7% reported adequate concentration, while all participants indicated that their work schedule affected memory, and 86.7% reported impacts on concentration.

Among the FSW group, 33.3% of the participants reported a history of otitis, 6.7% reported tinnitus, 20% experienced vertigo, and 66.7% were on medication. Comorbidities included isolated elevated cholesterol, elevated uric acid, and combinations of cholesterol with hypertension, steatosis, or thyroid disorders. Median sleep duration was 6 h (range: 5–7), increasing to 7 h (range: 5–9) on the night before assessment. Most participants reported good memory (93.3%) and concentration (73.3%), while only 26.7% noted schedule-related effects. None of the participants had undergone ear surgery. Overall, SW participants experienced greater subjective impacts of work schedule on memory and concentration, associated with shorter sleep duration, despite similar comorbidity prevalence between groups (see Table 2 and Table 3). 

SW group showed slightly better PTAs and high-frequency thresholds (HF Avg) bilaterally compared to FSW group; however, the differences were not statistically significant (Right ear: SW PTA 8.25 ± 4.93 dB and HF Avg 7.11 ± 5.17 dB vs. FSW PTA 9.08 ± 5.89 and HF Avg 8.78 ± 7.80; Left ear: SW PTA 7.33 ± 4.55 dB and HF Avg 7.67 ± 4.91 dB vs. FSW PTA 8.75 ± 6.27 and HF Avg 9.88 ± 7.88; *p* > 0.05). Similarly, mean speech intelligibility was higher in SW participants compared with fixed-schedule workers in both quiet (85.73 ± 7.70% vs. 84.53 ± 10.04, respectively) and noise (76.33 ± 10.78% vs. 74.87 ± 10.45, respectively) conditions. Regarding mean listening effort, this group obtained a percentage of 59.60% (±25.89%), with a median of 66%, whereas the FSW group exhibited a mean percentage of 52.93 ± 27.57. Although higher scores indicate lower listening effort, no statistically significant differences were observed between the two groups. (*p* > 0.05) (see Table 4). 

SW participants demonstrated higher mean scores than FSW participants in visuospatial/executive function (4.47 ± 0.64 vs. 3.80 ± 1.15), attention (5.53 ± 0.64 vs. 5.13 ± 0.74), memory recall (2.33 ± 1.54 vs. 1.27 ± 1.22), memory index (10.47 ± 2.72 vs. 7.67 ± 3.09), and overall MoCA (25.53 ± 2.29 vs. 23.40 ± 2.38). 

Statistically significant differences were observed in the total MoCA score (*p* = 0.033), memory recall (*p* = 0.049), and memory index (*p* = 0.015). (see Table 5). Subsequent analysis with ANCOVA showed that the effect of work schedule on cognitive performance remained significant after adjusting for age (F(1,27) = 6.976, *p* = 0.014, partial η^2^ = 0.205). Estimated marginal means indicated that shift workers (M = 25.58) scored significantly higher than fixed-schedule workers (M = 23.35), with a mean difference of 2.23 (*p* = 0.014, Bonferroni-adjusted). These findings confirm that the observed differences are not attributable to age differences between groups.

Correlation analyses revealed distinct patterns between shift workers and fixed-schedule workers. Among shift workers, right-ear measures showed the strongest associations with cognitive performance. Specifically, right-ear PTA was strongly correlated with the memory index (r = 0.742, *p* = 0.002) and moderately with memory recall (r = 0.582, *p* = 0.023). Right ear HF Avg also correlated with the memory index (r = 0.538, *p* = 0.039). In contrast, left-ear measures and speech intelligibility in either quiet or noise were not significantly associated with cognitive outcomes in this group. 

In fixed-schedule workers, right ear PTA was negatively correlated with orientation (r = −0.542, *p* = 0.037), and left ear HF Avg was inversely associated with total MoCA performance (r = −0.515, *p* = 0.049). Notably, speech intelligibility in quiet showed a strong positive correlation with orientation (r = 0.671, *p* = 0.006). Other measures, including speech intelligibility in noise and listening effort, showed only modest or non-significant associations. Table 6 and Table 7 summarize the main correlations between auditory measures and cognitive scores in shift workers and fixed-schedule workers, respectively. 

Given the relatively small sample size and the large number of comparisons, the risk of Type I error is increased. Although some differences and correlations reached nominal significance, these did not survive Bonferroni correction and therefore should be interpreted as exploratory. These preliminary findings warrant replication in larger samples.

## 4. Discussion

This study examined the associations between shift work and auditory–cognitive processing in middle-aged healthcare workers. Our findings highlight that, despite similar comorbidity profiles, shift workers experienced more pronounced subjective effects of their work schedule on memory and concentration and showed specific associations between auditory measures and cognitive performance. This pattern supports the notion that cumulative effects of shift work become evident in midlife. Shift work is well known to disrupt circadian rhythms, thereby increasing the risk of metabolic dysregulation, cardiovascular disease, and insulin resistance. Middle-aged adults may be particularly vulnerable due to declining physiological resilience and accumulated comorbidities [22,23]. In our sample, medication use and comorbidity prevalence were high in both groups, reflecting typical midlife health patterns.

Regarding sleep patterns, it was observed that they differed markedly. Even though mean sleep duration was similar between groups (SW group: ~6.3 vs. FSW group: 6.4 h), shift workers showed greater variability and slept significantly less on the night before testing (2.87 vs. 6.73h). In fact, only 40% of SW participants reported restorative sleep. By contrast, fixed-schedule workers reported more consistent sleep patterns. Interestingly, vertigo and tinnitus were slightly more frequent in this group, although not statistically significant, possibly reflecting age-related vestibular or auditory function [24,25]. 

Audiometric results were within normal limits (PTAs < 10 dB HL) in both groups. High-frequency thresholds and speech intelligibility were in accordance with hearing thresholds; however, intelligibility performance in noise was reduced, consistent with known midlife listening challenges. Listening effort was moderate in both groups (~60% in the SW group vs. ~53% in the FSW group). Unexpectedly, SW participants demonstrated slightly lower listening effort, which may reflect greater allocation of cognitive resources to memory-related tasks [26]; however, this difference was not statistically significant. 

Accordingly, shift workers tended to show higher memory and recall scores than fixed-schedule workers; however, these differences did not remain significant after correction for multiple comparisons. Therefore, these preliminary patterns may suggest enhanced preservation of specific cognitive domains but should be interpreted with caution given the small sample size and number of comparisons. 

Although exploratory, these results raise the possibility that middle-aged shift workers may develop compensatory strategies, such as enhanced executive control, attentional flexibility, and memory reliance, to maintain performance in complex auditory environments, such as healthcare facilities with background noise and overlapping speech. In fact, the literature suggests that these compensatory mechanisms are particularly relevant for women in the menopausal transition [27,28], who represented the majority of the sample (86.7%). However, it seems that complex environments associated with circadian rhythm disruption may be a potential factor for cognitive resilience during midlife. Nonetheless, it should be emphasized that chronic circadian disruption remains an established risk factor for cognitive decline [29,30], and more extensive and longitudinal studies should be performed. 

Additionally, auditory–cognitive correlations showed group-specific exploratory patterns. In the SW group, right-ear thresholds (PTA and HF Avg) were moderately to strongly positively associated with memory recall. These findings should be interpreted as preliminary and hypothesis-generating rather than confirmatory. However, this aligns with the well-documented right-ear advantage for verbal memory [30,31] and suggests that, together with reduced listening effort, sleep dysregulation, and working in challenging conditions, it may promote a reallocation of cognitive resources toward auditory memory, underscoring the interplay between auditory input and cognition in complex environments. FSW participants showed weaker associations, likely reflecting more stable circadian rhythms. Nonetheless, right-ear PTA and speech intelligibility in quiet were negatively and positively correlated, respectively, with the orientation domain. This pattern supports the role of age-related subtle auditory variations in spatial and temporal processing and highlights the impact of auditory factors on cognitive outcomes, even in relatively stable circadian contexts. 

Lastly, the prevalence of comorbidities such as hypercholesterolemia and thyroid problems was generally low and similar between shift workers and fixed-schedule workers, making major confounding effects unlikely. However, due to the small sample size, the observed group differences and correlations should be interpreted with caution. These findings are best understood as exploratory, providing hypotheses for future studies with larger samples and more stringent designs. Therefore, residual confounding cannot be excluded. Furthermore, unlike most previous studies, our analysis did not rely solely on the MoCA total score. In fact, we examined each cognitive domain separately. This methodological approach allowed for a more detailed characterization of potential differences in cognitive performance between groups, which may partly explain the divergence from prior findings. An additional source of bias that warrants consideration is the possibility that individuals with higher baseline cognitive abilities may have preferentially self-selected or been assigned to shift work roles, which are often more cognitively demanding. This could create a pre-existing selection advantage rather than a shift-work-induced cognitive benefit. However, the previous literature highlights educational attainment as a key determinant of cognitive reserve, which may contribute to baseline differences in cognitive performance [32,33,34]. In our study, both groups were predominantly composed of individuals with high educational levels, worked in the same hospital, and lived in similar socioeconomic contexts, which reduces the likelihood of baseline cognitive differences.

## 5. Conclusions

Despite the well-known association between shift work, sleep disruption, and adverse health outcomes, this exploratory study found preliminary patterns suggesting that shift work may be associated with preserved cognitive performance in memory and executive domains in middle-aged healthcare workers. Cognitive reserve, long-term occupational experience, and adaptive strategies may contribute to buffering against deficits, particularly in acoustically complex environments. However, these observations did not remain significant after adjustment for multiple comparisons and should therefore be interpreted with caution. The small sample size, predominance of women, and cross-sectional design limit the generalizability of these findings and preclude causal inference. Larger, longitudinal studies are needed to clarify whether such patterns reflect real and permanent adaptive mechanisms. 

## Figures and Tables

**Table 1:** Distribution of healthcare professions by work schedule.

	SW Group N (%)	FSW Group N (%)
Nurse	11 (57.9%)	8 (42.1%)
Health Care Assistant	4 (50.0%)	4 (50.0%)
Other Clinical Professionals	0 (0.0%)	2 (100.0%)
Clinical Secretary	0 (0.0%)	1 (100.0%)

**Table 2:** Prevalence of health conditions according to work schedule.

		SW Group		FSW Group	
		N	%	N	%
Otitis	Yes	5	33.3%	5	33.3%
	No	10	66.7%	10	66.7%
Tinnitus	Yes	0	0.0%	1	6.7%
	No	15	100.0%	14	93.3%
Vertigo	Yes	1	6.7%	3	20.0%
	No	14	93.3%	12	80.0%
Medication	Yes	8	53.3%	10	66.7%
	No	7	46.7%	5	33.3%
Chronic Condition	Cholesterol	5	33.3%	4	26.7%
	Uric Acid	-	-	2	13.3%
	Others	3	20.0%	4	44.4%
	Thyroid Problems	1	6.7%	1	6.7%
	Hypertension	4	26.7%	2	13.3%
	Steatosis	-	-	1	6.7%
	Diabetes	2	13.3%	-	-
	Sleep Apnea	1	6.7%	-	-
	Obesity	1	6.7%	-	-

**Table 3:** Sleep patterns and cognitive effects according to work schedule.

		SW Group	FSW Group
Sleep Hours	Mean (SD)	6.33 (1.11)	6.4 (0.63)
	Median	6.0	6.0
Sleep Hours (night before evaluation)	Mean (SD)	2.87 (2.42)	6.73 (1.10)
	Median	3.0	7.0
Rest Well	Yes N (%)	6 (40.0%)	3 (20.0%)
	No N (%)	9 (60.0%)	12 (80.0%)
Good Memory	Yes N (%)	9 (60.0%)	14 (93.3%)
	No N (%)	6 (40.0%)	1 (6.7%)
Memory Impact	Yes N (%)	15 (100.0%)	4 (26.7%)
	No N (%)	0 (0.0%)	11 (73.3%)
Good Concentration	Yes N (%)	10 (66.7%)	11 (73.3%)
	No N (%)	5 (33.3%)	4 (26.7%)
Concentration Impact	Yes N (%)	13 (86.7%)	4 (26.7%)
	No N (%)	2 (13.3%)	11 (73.3%)

**Table 4:** Results of auditory measures according to work schedule.

		SW Group	FSW Group
Right Ear: PTA	Mean (SD)	8.25 (4.93)	9.08 (5.89)
	Median	7.50	8.75
Right Ear: HF Avg	Mean (SD)	7.11 (5.17)	8.78 (7.80)
	Median	6.67	6.67
Left Ear: PTA	Mean (SD)	7.33 (4.55)	8.75 (6.27)
	Median	7.50	7.50
Left Ear: HF Avg	Mean (SD)	7.67 (4.91)	9.88 (7.88)
	Median	6.67	8.30
Speech Intelligibility in Quiet	Mean (SD)	85.73 (7.70)	84.53 (10.04)
	Median	86.00	88.00
Speech Intelligibility in Noise	Mean (SD)	76.33 (10.78)	74.87 (10.45)
	Median	78.00	73.00
Listening Effort	Mean (SD)	59.60 (25.89)	52.93 (27.57)
	Median	66.00	66.00

**Table 5:** MoCA scores by group.

	SW Group		FSW Group
Visuospatial/Executive Function (Maximum Score: 5)	Mean (SD)	4.47 (0.64)	3.80 (1.15)
	Median	5.00	4.00
Naming (Maximum Score: 3)	Mean (SD)	2.73 (0.46)	2.93 (0.26)
	Median	3.00	3.00
Attention (Maximum Score: 6)	Mean (SD)	5.53 (0.64)	5.13 (0.74)
	Median	6.00	5.00
Language (Maximum Score: 3)	Mean (SD)	2.47 (0.64)	2.47 (0.52)
	Median	3.00	2.00
Abstraction (Maximum Score: 2)	Mean (SD)	2.00 (0.00)	2.00 (0.38)
	Median	2.00	2.00
Memory Recall (Maximum Score: 5)	Mean (SD)	2.33 (1.54)	1.27 (1.22)
	Median	3.00	1.00
Memory Index (Maximum Score: 15-excluded from total MoCA score)	Mean (SD)	10.47 (2.72)	7.67 (3.09)
	Median	11.00	8.00
Orientation (Maximum Score: 6)	Mean (SD)	6.00 (0.00)	5.80 (0.41)
	Median	6.00	6.00
MoCA (Maximum Score: 30)	Mean (SD)	25.53 (2.29)	23.40 (2.38)
	Median	26.00	23.00

**Table 6:** Correlations between auditory measures and cognitive scores in SW group (N = 15).

		Memory Recall	Memory Index	MoCA
Right Ear: PTA	r	0.582	0.742	0.422
	*p*	0.023	0.002	0.117
Right Ear: HF Avg	r	0.430	0.538	0.169
	*p*	0.110	0.039	0.546

**Table 7:** Correlations between auditory measures and cognitive scores in FSW group (N = 15).

		Naming	Attention	Orientation	MoCA
Right Ear: PTA	r	−0.186	−0.358	−0.542	−0.400
	*p*	0.507	0.190	0.037	0.140
Left Ear: HF Avg	r	−0.125	−0.482	−0.466	−0.515
	*p*	0.658	0.069	0.080	0.049
Speech intelligibility in quiet	r	0.253	0.134	0.671	0.474
	*p*	0.363	0.634	0.006	0.074

## Data Availability

The data presented in this study are available on request from the corresponding author. The data are not publicly available due to privacy and ethical restrictions.
